# Respiratory and Other Health Effects Reported in Children Exposed to the World Trade Center Disaster of 11 September 2001

**DOI:** 10.1289/ehp.11205

**Published:** 2008-06-18

**Authors:** Pauline A. Thomas, Robert Brackbill, Lisa Thalji, Laura DiGrande, Sharon Campolucci, Lorna Thorpe, Kelly Henning

**Affiliations:** 1 New Jersey Medical School–University of Medicine and Dentistry of New Jersey, Newark, New Jersey, USA; 2 Agency for Toxic Substances and Disease Registry, U.S. Department of Health and Human Services, Atlanta, Georgia, USA; 3 RTI International, Chicago, Illinois, USA; 4 New York City Department of Health and Mental Hygiene, New York City, New York, USA; 5 Bloomberg Foundation, New York City, New York, USA

**Keywords:** air pollution, asthma, postdisaster health assessment in children, respiratory health, World Trade Center disaster

## Abstract

**Background:**

Effects of the World Trade Center (WTC) disaster on children’s respiratory health have not been definitively established.

**Objective:**

This report describes respiratory health findings among children who were < 18 years of age on 11 September 2001 (9/11) and examine associations between disaster-related exposures and respiratory health.

**Methods:**

Children recruited for the WTC Health Registry (WTCHR) included child residents and students (kindergarten through 12th grade) in Manhattan south of Canal Street, children who were south of Chambers Street on 9/11, and adolescent disaster-related workers or volunteers. We collected data via computer-assisted telephone interviews in 2003–2004, with interview by adult proxy for children still < 18 years of age at that time. We compared age-specific asthma prevalence with National Health Interview Survey estimates.

**Results:**

Among 3,184 children enrolled, 28% were < 5 years of age on 9/11; 34%, 5–11 years; and 39%, 12–17 years. Forty-five percent had a report of dust cloud exposure on 9/11. Half (53%) reported at least one new or worsened respiratory symptom, and 5.7% reported new asthma diagnoses. Before 9/11, age-specific asthma prevalence in enrolled children was similar to national estimates, but prevalence at interview was elevated among enrollees < 5 years of age. Dust cloud exposure was associated with new asthma diagnosis (adjusted odds ratio = 2.3; 95% confidence interval, 1.5–3.5).

**Conclusions:**

Asthma prevalence after 9/11 among WTCHR enrollees < 5 years of age was higher than national estimates, and new asthma diagnosis was associated with dust cloud exposure in all age groups. We will determine severity of asthma and persistence of other respiratory symptoms on follow-up surveys.

An estimated 25,000 children < 18 years of age were living or attending school in lower Manhattan near the World Trade Center (WTC) on 11 September 2001 (9/11), and tens of thousands more were in the path of the plume of building debris and smoke, close enough to inhale particulates and toxic substances (U.S. Census Bureau 2000; U.S. Department of Education 2006). The effects of these exposures on children’s health have not been definitively established.

Physical health consequences described in adult survivors of the disaster have included injuries sustained while fleeing the disaster (Boodram et al. 2002); pulmonary damage in rescuers, ironworkers, and others who had intense exposure to disaster-related fires (Asarnow et al. 2003; Banauch et al. 2003; Landrigan et al. 2004; Prezant et al. 2002; Skloot et al. 2004); and exacerbations of respiratory illness, including self-reported asthma (Brackbill et al. 2006; Fagan et al. 2002; Lin et al. 2005; Reibman et al. 2005; Wheeler et al. 2007). Pollutants included particulates blown from collapsing buildings and resuspended during cleanup, shown in laboratory studies to cause respiratory irritation; and a variety of toxic compounds from fires that burned until 20 December 2001, including polycyclic aromatic hydrocarbons, volatile organic compounds, lead, dioxin, and particulates [Butt et al. 2004; Gavett et al. 2003; Jeffery et al. 2003; Lioy et al. 2002; McGee et al. 2003; New York City Department of Health and Mental Hygiene (NYCDOHMH) and Agency for Toxic Substances and Disease Registry (ATSDR) 2002; Payne et al. 2004; Pleil et al. 2004; Service 2003].

Two studies suggested exacerbation of asthma among children during the autumn of 2001 (Szema et al. 2004; Wagner et al. 2005). Szema et al. (2004) examined asthma status in the year before and after 9/11 in 205 children of Chinese descent who had previously diagnosed asthma. They found that children living near the WTC site had increased asthma-related clinic visits during the year after 9/11, but rescue inhaler use was greater in children who lived farther away. In mid-2002, Wagner et al. (2005) conducted a mail survey and medical record review of adults and children with known asthma receiving Medicaid-managed care. Although they noted increased asthma exacerbation among persons living near the WTC, data were not provided specifically for children in that group. No follow-up has been reported.

After the 9/11 attacks in New York City, the WTC Health Registry (WTCHR) was established to evaluate short- and long-term physical and psychological effects of the disaster. The registry enrolled persons most likely to have been heavily exposed to traumatic events and air pollutants, including 3,184 children < 18 years of age on 9/11 (Brackbill et al. 2006). The purpose of this report is to present baseline data on respiratory health, and to examine associations between disaster-related exposures and health, among the children enrolled in the WTCHR, as reported on initial registry interviews of parents and adolescents conducted September 2003 through November 2004.

When the registry was undertaken, it was already known that all but two of the 25,000 children who were at home or in school in lower Manhattan had been successfully evacuated from downtown Manhattan (Schwartz et al. 2002). Two youths 15–19 years of age died in WTC buildings on 9/11 (NYCDOHMH, unpublished data). Hundreds of other children lost parents in the disaster. These children are the subject of other work and are not included in the WTCHR unless they themselves were in the vicinity of the disaster.

## Materials and Methods

In this analysis, we describe information collected on persons who were 0–17 years of age on 9/11. Interviews were conducted between 5 September 2003 and 20 November 2004. Interviews of those < 18 years at the time of the interview were by proxy; youths who were no longer minors (i.e., who were ≥ 18 years of age at the time of the interview) answered the interview questions themselves. Recruitment and data collection methods for the registry overall are described by Brackbill et al. (2006) and Dolan et al. (2006). In the following, we describe methods specific for children.

### Eligibility, identification, and recruitment

**Figure 1:**
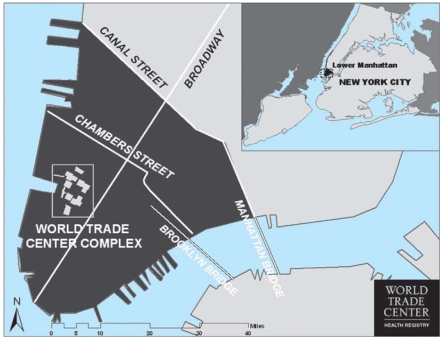
Map showing lower Manhattan in New York City: site of WTC, Canal Street (northern boundary of recruitment for WTCHR), and Chambers Street.

Children eligible to enroll or to be enrolled in the WTCHR had at least one of the following criteria: residence or school in Manhattan south of Canal Street, presence south of Chambers Street on the morning of 9/11, or work in disaster-related rescue or recovery. Figure 1 is a map of lower Manhattan, showing the location of the WTC site, Chambers Street, and Canal Street.

Children and teenagers who were < 18 years of age on 9/11 were actively recruited for the WTCHR through their parents, telephone calls to area residents, and active outreach to schools and community organizations. An initial listing of names associated with approximately 24,000 households south of Canal Street was purchased from a commercial company (Genesys Sampling, Fort Washington, PA). A letter was sent to each home, followed by a telephone call from a trained interviewer. The letter included the registry website and toll-free number. A listing was developed of 37 schools south of Canal Street in Manhattan, including child care centers, nursery schools, and public and private schools with grades kindergarten through 12th grade (K–12). Each school was contacted by mail and telephone, and registry staff gave presentations about the project to teacher and parent groups. Nine of the private schools provided lists of student names. The New York City Department of Education mailed a letter explaining and endorsing the registry project to families of 12,600 public school children (Murphy et al. 2007). Some children were identified during interviews with adults in their households. Additional outreach was made to reach youths who had passed their 18th birthdays, through e-mails to colleges and high school alumni groups.

Parents could enroll children, and youths ≥ 18 years of age could enroll themselves, by calling a toll-free number or preregistering on the WTCHR website.

### Interview methods

All WTCHR data were collected by computer-assisted interview. The interview instrument was translated into and bilingual interviewers were available to conduct interviews in English, Spanish, Mandarin, and Cantonese. Interviews in other languages were conducted by three-way telephone connection with a professional language service center.

### Content of interview

The initial WTCHR interview is available online (Pulliam et al. 2006). It included determination of eligibility, demographics, disaster-related exposures, injuries sustained on 9/11, physical health symptoms and conditions before and after 9/11, and mental-health–related symptoms during the month before the interview. The interview did not include questions regarding whether respondents sought treatment, nor were medical records reviewed or medical examinations conducted. We compared age-specific asthma prevalences before 9/11 and at the time of the interview with rates of self-reported asthma from the 2003 National Health Interview Survey (NHIS) (Centers for Disease Control and Prevention 2003). Unlike the WTCHR interview, the NHIS interview took place face to face, not over the telephone. However, the question to determine child asthma prevalence was identical to that in the WTCHR: “Have you EVER been told by a doctor or other health professional that you [your child] had asthma?”

The interview for children and youths 12–17 years of age on 9/11 included questions on smoking also taken from the NHIS adult survey. The questions were “Have you [or, if by proxy, the child or youth] ever smoked at least 100 cigarettes?” and, if yes, “Are you [is he or she] still smoking?”

For children < 18 years of age at time of interview, the interview included eight questions about stress symptoms during the 4 weeks before the interview, with reference to 9/11, derived from a post-9/11 survey of mental health effects on New York City school children (Hoven et al. 2005). For the WTCHR, we considered the child to have possible post-traumatic stress symptoms at the time of interview if the parent answered “yes” to at least six of the eight questions.

### Exposure information

Interviews asked whether the child *a*) was caught in the dust and debris cloud resulting from collapsing buildings, *b*) personally witnessed disturbing disaster-related events (an airplane hitting a WTC tower, a building collapsing, people running away, someone who was injured or killed, or people falling or jumping from the WTC towers), *c*) was evacuated from home or school, *d*) worked in rescue/recovery (asked of those ≥ 12 years of age), and *e*) spent time at home or in school in lower Manhattan from 9/11 to 20 December 2001, while the fires burned.

#### Definition of dust cloud exposure

An evaluation of where people were located, at the time they reported being caught in the dust and debris cloud from collapsing buildings, revealed locations throughout New York City, including areas distant from the WTC site where there was no documented intense dust cloud. To categorize this exposure as accurately as possible, we created a second variable: “dust cloud exposure with eye irritation.” People who met criteria for this exposure were those who reported both *a*) being caught in the dust cloud and *b*) eye injury or irritation on 9/11, with the idea that eye irritation on 9/11 was very likely caused by intense exposure to the debris thrown out as buildings collapsed.

#### Time spent in lower Manhattan

We calculated an estimate of the time spent in lower Manhattan for residents and students. For resident children who attended school in lower Manhattan, we assumed exposure to be 24 hr/day from 9/11 or, if they evacuated, from the date they returned home until 20 December 2001, when the WTC fires were declared over. For resident children who attended school outside of the area, we subtracted 8 hr/day for school days (5 days/week minus school holidays). For students who were not residents of lower Manhattan, we assumed exposure to be 8 hr/day on school days (Monday through Friday, minus school holidays).

#### Distance of home or school from WTC site

We geocoded residential addresses and computed straight-line distance to the center of the WTC site using ArcView version 8.3 (ESRI, Redlands, CA). We also geocoded addresses of schools south of Canal Street and computed distance in feet to WTC epicenter.

#### Enrollment rates by population group

We calculated the percentage of eligible residents 0–17 years of age on 9/11, who enrolled in the WTCHR, based on population estimates by census-block–level population (U.S. Census Bureau 2000). To calculate percentage of students enrolled in the registry, we obtained school census data for 2001 (Murphy et al. 2007).

### Statistical analysis

Descriptive analysis included frequency by enrollment category, disaster-related exposures, and major self-reported health findings. We used a separate bivariate logistic regression model for newly diagnosed asthma. We computed crude odds ratios (ORs) for demographic variables and exposures. We considered covariates in a final multivariate logistic model if their *p*-values were < 0.05 in the bivariate logistic model. We selected final models based on models that achieved the highest likelihood ratio chi-square and were conceptually appropriate. We used SAS version 9.1 (SAS Institute Inc., Cary, NC) for computing frequency distributions, adjusted ORs, and 95% confidence intervals (CIs). Other model variables not described above included race/ethnicity [based on two questions: “Are you Hispanic or (Latino/Latina)?” and “Which of the following would you say is your race? You may select more than one category.”]; injured on 9/11 (based on the question: “On 9/11, did you have any of the following injuries as a result of World Trade Center terrorist attack?”). Further descriptions of variables, including the baseline questionnaire, are reported by Pulliam et al. (2006).

This study was approved by the institutional review boards of both the NYCDOHMH and the Centers for Disease Control and Prevention. Participants provided verbal informed consent.

## Results

### Characteristics of enrollees

Of the 71,437 people enrolled in the WTCHR, 3,251 were children < 18 years of age on 9/11. We excluded 67 children because they met none of the WTCHR eligibility criteria. Table 1 lists the demographic characteristics of the remaining 3,184. Half (49%) were boys, 47% were non-Hispanic white, 19% Asian, 19% Hispanic, and 8% non-Hispanic black. At the time of exposure, 27.5% were < 5 years of age, 33.7% were 5–11, and 38.9% were 12–17. Not shown in Table 1, of those children who were < 18 years of age on 9/11, 614 (19.3%) were ≥18 years of age at the time of the initial interview. For this group, the youth, not the parent, answered the interview questions. Most interviews (89%) were conducted in English, 8.9% in a Chinese dialect (either Cantonese or Mandarin), and 1.5% in Spanish. The telephone operator translator was used for only 12 interviews.

Overall enrollment among 8,310 children living south of Canal Street in 2001 was 21%. The percentage of eligible children enrolled was equal for boys and girls, slightly higher for younger children, and higher for whites and Hispanics than for blacks and Asians. Estimated enrollment among eligible children attending school south of Canal Street was 13.8% for preschool and 11.3% for children enrolled in grades K–12.

Most children (69%) were residents of lower Manhattan south of Canal Street, half of whom also attended school in that area. Another 26% were lower Manhattan students who lived elsewhere in New York City. One hundred thirty adolescents (4% of the 3,184 enrollees) had worked in rescue and recovery activities; most of these youths were 16–17 years of age on 9/11, and 45% were also students or residents in lower Manhattan. Most had been involved in volunteer activities with agencies such as the Red Cross.

**Figure 2:**
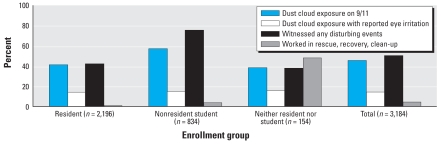
Exposures related to the WTC 9/11 disaster, reported for 3,184 children enrolled in WTCHR: percent exposed by enrollment group. “Neither resident nor student” indicates 74 youths who worked in rescue/recovery and 80 passers-by south of Chambers Street on the morning of 11 September 2001. “Witnessed any disturbing event” included personally seeing an airplane hitting the WTC, people falling or jumping from the WTC, buildings collapsing, people running away from a cloud or smoke, or people injured or killed.

Figure 2 shows the proportion of children in each eligibility group for whom there was a report of dust cloud exposure, witnessing disturbing events, or work in rescue, recovery, or cleanup activities. Nearly half of the children (45%) were reported to have been caught in the dust cloud that resulted from the collapsing buildings on the morning of 9/11, including 459 (14%) for whom eye injury or irritation was also reported. Half of the children (50%) were reported to have witnessed at least one disturbing disaster-related event.

Among children who resided south of Canal Street, 1,302 (59.3%) had to evacuate from their homes (data not shown). Nearly all (89%) child evacuees returned home before the end of 2001. Among the 1,923 youths < 18 years of age on 9/11 who were enrolled in schools (prekindergarten, K–12) south of Canal Street, most (87.6%) evacuated from their schools. Of those, 59.5% returned in 2001 while the fires still burned.

The 2,196 resident children were from 1,829 households. Thirty percent were from households with no adult enrolled in the WTCHR (534 households). A history of smoking was reported for 116 (8%) of those ≥ 12 years of age.

### Health findings

Table 2 shows the frequency of symptoms and health outcomes by age. Eye irritation or eye injury on 9/11 was reported in 22% of children. Injuries on 9/11 (sprains, lacerations, burns, broken bones, or concussions) were reported for 3%. Half the children had at least one new or worsened respiratory symptom at some time after 9/11, including shortness of breath, cough, sinus problems, throat irritation, or wheezing. Proportional distribution of new and worsened wheezing showed higher rates in the younger children: 29% in those 0–4 years of age, 26% in children 5–11, and 23% in children 12–17. Not shown, higher rates of new or worsened wheezing were reported in black and Hispanic children (38% and 34%, respectively) compared with whites and Asians (20% and 23%, respectively). Information was not collected on whether these symptoms persisted after December 2001, when fires were extinguished and dust from transport of debris diminished.

In answer to the question “Have you ever been told by a doctor or other health professional that you [your child] had asthma?” 19% of interviews indicated “yes” for diagnoses made as of the time of the interview. For 180 children (6%), the diagnosis of asthma occurred after 9/11. Half of new-onset asthma diagnoses were in children < 5 years of age on 9/11. Not shown in Table 2, the prevalence of asthma by race/ethnicity followed a pattern similar to wheezing: Report of asthma diagnosis was lowest in white and Asian children [14.4% (95% CI, 12.7–16.2%) and 15.9% (13.2–19.0%), respectively] and twice as high in black and Hispanic children [27.9% (22.8–33.7%) and 30.1% (26.7–33.8%), respectively].

Table 3 shows data on reported prevalence of asthma by age on 9/11 and prevalence by age at interview. Age-specific prevalence of diagnosed asthma in WTCHR children before 9/11 was comparable with the prevalence reported for U.S. children overall in the 2003 NHIS (Centers for Disease Control and Prevention 2003). In contrast, at the time of the interview, 2–3 years after 9/11, age-specific asthma prevalence was higher than the NHIS rates: Prevalence among those 2–4 years of age by the time of the interview was 16% (95% CI, 12.4–19.7%), versus 9.1% (95% CI, 7.8–10.2%) in the NHIS. Among those 5–11 years of age at the time of the interview, prevalence was 18.7% (95% CI, 16.5–21.0%), versus 14.0% (95% CI, 13.0–15.0%) in the NHIS, and for those 12–17 years of age, 19.0% (95% CI, 16.6–21.4%), versus 14.6% (95% CI, 14.0–16.0%) in NHIS. Because rates of lifetime asthma are somewhat higher for the Northeast region of the United States, we also compared the post-9/11 rates with NHIS rates for the Northeast region only. Age-specific prevalence of asthma at interview remained higher than regional NHIS rates only for the children who were < 5 years of age at the time of the interview.

### Factors associated with a new diagnosis of asthma

Table 4 shows the associations between new diagnoses of asthma, registrant characteristics, and WTC-related exposures. In univariate analyses, young age on 9/11 had the strongest association with a new diagnosis of asthma after 9/11 (< 5 years of age, 10.8%, vs. 12–17 years, 2.8%; OR = 4.2; 95% CI, 2.8–6.3). Hispanic and Asian ethnicity, and being caught in the dust cloud were also associated with new asthma diagnosis on univariate analysis. The association was strongest for those who reported being caught in the dust cloud and having eye irritation or injury: 8.6% vs. 4.4% compared with those reporting neither (OR = 2.0; 95% CI, 1.4–3.0).

In multivariable analysis, children of Asian or Hispanic ethnicity were twice as likely as other children to have been diagnosed with new asthma after 9/11 (7.7% of Asian children and 8.0% of Hispanics had a new diagnosis of asthma, compared with 4.1% of whites). Being caught in the dust cloud with eye irritation was associated with a new diagnosis of asthma (adjusted OR = 2.2; 95% CI, 1.5–3.4). When the multivariable model was restricted to children < 5 years of age on 9/11, the associations were similar to those for the group overall. For all age groups, the percentage of children with new asthma diagnosis was higher among those with reported exposure to the dust cloud on 9/11. However, for the youngest group, prevalence was higher than NHIS rates even among those with no reported dust cloud (data not shown).

In univariate and multivariable analysis, distance of home or school from the WTC site, and duration of time spent in lower Manhattan while the fires burned were not associated with new asthma diagnosis (Table 4).

## Discussion

In this report we describe disaster-related exposures and physical and respiratory health reported on an initial interview survey among 3,184 children enrolled in the WTCHR. Children < 5 years of age at the time of interview had a higher than expected prevalence of reported asthma after 9/11, and new diagnosis of asthma in all age groups was significantly associated with exposure to the dust cloud on 9/11. The OR for the association was increased when we restricted the outcome to those with dust cloud exposure plus eye irritation. More than half of the children had new or worsened respiratory symptoms at some time after 9/11, but the importance of this is not yet known because duration and severity of these symptoms were not determined on the initial interviews. Few children (3%) sustained direct injuries on 9/11.

The high proportion of children with new or worsened respiratory symptoms parallels findings reported for adults who lived or worked in lower Manhattan in the fall of 2001 (Brackbill et al. 2006; Lin et al. 2005; Reibman et al. 2005). Follow-up of the children is needed to determine severity and duration of the symptoms reported. In adults, many symptoms abated when the smoke and dust cleared after December 2001 (Reibman et al. 2005).

More severe pulmonary injury has been reported in workers who had close and intense exposure to dust and burning debris (Banauch et al. 2003; Landrigan et al. 2004; Skloot et al. 2004; Wheeler et al. 2007). Although few residents and school children had such intense exposures, nearly half of our cohort had a report of dust cloud exposure, and this was associated with new asthma diagnosis. The dust cloud was a quickly expanding mass of debris that included, among other toxics, large amounts of alkaline pulverized cement, a mucous membrane irritant. We did not find an association of new asthma diagnosis with length of time spent in lower Manhattan from 9/11 to 20 December 2001, when air pollution continued because of disaster-related fires and cleanup activities that contributed to airborne particulates. However, we cannot rule out the role of these exposures, because our estimate of time in lower Manhattan was crude, and we lacked precise information on characteristics of the children’s homes and schools (e.g., were windows open and facing truck routes, were effective filters installed).

Others have reported exacerbation of previously diagnosed asthma among adults and children in New York City after 9/11 (Fagan et al. 2002; Szema et al. 2004; Wagner et al. 2005). Fagan et al. (2002) reported that among 13% of Manhattan adults with asthma interviewed by telephone 5–9 weeks after 9/11, 27% reported an exacerbation. Two studies reported post-9/11 asthma exacerbations among children with previously diagnosed asthma (Szema et al. 2004; Wagner et al. 2005). Szema et al. (2004) used data from one clinic in Manhattan’s Chinatown to compare children who lived within 5 miles of the WTC site with children who lived farther away. Children living < 5 miles from the WTC site showed a larger increase in clinic visits for asthma after 9/11, but use of rescue inhaler was greater in children at a distance. The study was limited by small sample size and use of residence distance from the WTC site as a proxy for actual exposure (Matte and Mostashari 2004). The study did not determine whether additional asthma-related care was obtained elsewhere; thus, data may be missing, especially for the “controls,” who lived farther away. Wagner et al. (2005) examined adults and children with known asthma receiving Medicaid-managed care, using data from a mail survey and medical record review conducted in mid-2002. Overall, 21.7% of 16,629 eligible individuals 5–56 years of age responded to the survey, with lower response (19%) among parents of children 5–17 years of age. Although they noted increased asthma exacerbation among persons living near the WTC, data were not provided specifically for children included in that group (Wagner et al. 2005). None of these studies reported on new diagnosis of asthma after 9/11.

Both environmental and psychological factors can trigger asthma and wheezing (Eder et al. 2006; Wright et al. 1998). Our analysis found no association between reported stress and new-onset asthma; however, the WTCHR had very limited psychological data on children, and these initial WTCHR interviews were done 2–3 years after the event, measuring psychological stress at the time of interview, not at the time of the onset of asthma.

In our cohort, the baseline age-specific prevalence of asthma before 9/11 was comparable with published data for the United States and the Northeast United States, but at the time of interview, 2–3 years after 9/11, it was higher in the youngest group, children 2–4 years of age (Centers for Disease Control and Prevention 2003). Childhood asthma normally manifests during the first 5 years of life, often after exacerbation from an environmental irritant, allergen, or infection (Eder et al. 2006; Peden 2003; Pope 2000; Wong et al. 2004). It is possible that the intense exposures on 9/11 and during the ensuing months caused exacerbations in some children with a predisposition to asthma, resulting in increased detection and diagnosis at earlier ages than might otherwise have occurred. Consideration must also be given to the fact that children and infants are among the most susceptible to many air pollutants, in part because children’s lung development continues to adulthood (Kim 2004). Eighty percent of alveoli are formed postnatally, and ongoing lung growth and development continues through age 18 (Gauderman et al. 2004; Kim 2004). Although chronic exposure to air pollution has been associated with decreased pulmonary function at age 18 (Gauderman et al. 2004), no such data are available on short-term or disaster-related exposures (Gauderman et al. 2004; Kim 2004). The effect of outdoor air pollution on new development of asthma is not clear (Beasley et al. 2003; Kim 2004). In one prospective study, the risk of developing asthma was related to pollution only in children with high ozone exposure combined with vigorous exercise (McConnell et al. 2002).

Only long-term follow-up of the children enrolled in the WTCHR will determine whether they have a sustained increase in asthma prevalence. For example, if the higher rate in very young children is attributable to *de novo* disease, then the increased prevalence will continue as this cohort ages. On the other hand, if the higher rate is attributable to earlier exacerbation and diagnosis in children with a predisposition for asthma, the prevalence will level off to the overall population-wide prevalence rates at older ages.

We also found associations between new asthma diagnosis and ethnicity. White and Asian children had lower asthma prevalence than did black and Hispanic children, both before and after 9/11, although after 9/11, Asian and Hispanic children had the highest rates of new asthma diagnosis: 8% for each, compared with 4.4% for whites. Descriptive studies of asthma prevalence have shown high variability among subsets of Hispanics and Asians in the United States, including higher rates in some Hispanics and lower rates in Asian children (Davis et al. 2006; Lara et al. 2006). Asthma is thought to be influenced by many factors, including physical and social environment, genetics, and health-seeking patterns (Eder et al. 2006; Hunninghake et al. 2006). Information on pre-9/11 environmental influences for the WTCHR cohort is not available, but these influences likely play a role in the differences we observed.

It is also possible that ethnic differences in health care access and location of care influenced asthma diagnosis. Prior studies have shown that children are more likely to receive asthma diagnoses in emergency department settings than in primary care offices (Akinbami et al. 2005). Health-seeking behaviors differ by ethnicity; for example, rates of emergency department use and hospitalization differ by age and race (Grant et al. 1999). The WTCHR did not collect data on where and how the children were diagnosed, and it is likely that children of different ethnicities were receiving pediatric care in different settings. Black and Hispanic children also had much higher prevalence of increased or worsened wheezing after 9/11, suggesting possible missed opportunities for diagnosing asthma in these children.

### Limitations

Information on children’s respiratory health from the WTCHR interviews provides important baseline measures for a cohort that will be followed prospectively. However, these data have several limitations.

The voluntary nature of enrollment creates potential for bias. Parents who were especially worried, or whose children had been ill, may have been more likely to enroll in the WTCHR; for example, parents of children newly diagnosed with asthma might have been concerned that disaster-related exposures were the cause and might have been motivated to enroll these children. However, this cannot account for the strong association with dust cloud exposure, found in all ages, and with an even stronger association for those who reported both dust cloud exposure and eye irritation.

Reporting bias may have been introduced by using parent proxies for child interviews, although some data suggest good correlation between adolescents and parents on survey questions asking about prior diagnosis of asthma (Hedman et al. 2005; Magzamen et al. 2005).

Recall bias may influence responses 2–3 years after an event, although work after other disasters suggests that during such remarkable life episodes, memory is acute and well preserved (Bradburn et al. 1987). It is not known whether this applies to parents reporting for their children.

The WTCHR interview collected information on a variety of new or worsened physical health symptoms after 9/11, but not on how soon after 9/11 those symptoms first occurred or on their duration or severity. We did not determine asthma-related impairment. Physical symptoms and diagnoses were self-reported, not confirmed with medical record review or medical evaluation. Nonetheless, the question “Have you ever been told by a doctor or other health professional that you [your child] had asthma?” is considered reliable in surveys to study the epidemiology of asthma (Centers for Disease Control and Prevention 2003; Eder et al. 2006; Lara et al. 2006; Magzamen et al. 2005). This standardized question allows comparison with other surveys. Further study of the WTCHR cohort will require more detailed questions about frequency and severity of symptoms, and diagnostic tests.

The survey did not collect information on co-factors for asthma, including allergic status of the children and exposure to environmental tobacco smoke. Smoking histories, collected on children who were 12–17 years of age on 9/11, may be underreported by both parents as well as teens ≥ 18 years of age who answered their own interviews. We included smoking in the multivariable analysis of factors associated with new asthma diagnosis, but numbers reporting smoking were very small.

We were not able to adjust our analysis for cases where multiple children were enrolled from single households; instead, we gave each child equal weight in the analysis. Further, we did not collect data on the number of children from any household who were not enrolled.

Finally, our data on intensity of exposures to the dust cloud and time spent in lower Manhattan are limited because of the deliberate nature of the initial interview as a screening tool, to identify broad categories of exposure.

The strength of the WTCHR data is 2-fold. Despite the voluntary nature of enrollment, the registry constitutes the largest sample of child subjects exposed to the events of 9/11. The collection of exposure data allows for stratification of risk and enables examination of outcomes by exposure (e.g., presence in the dust and debris cloud or not, distance of school or home from WTC site). For some exposures, data are available to examine dose effect. Exposure registries do not routinely collect concurrent data from deliberately sampled unexposed persons, because no clearly stated hypothesis or defined outcome suggests what characteristics the comparison group should have (Goldhaber et al. 1983; NYCDOHMH and ATSDR 2004).

The WTCHR, with a large number of enrollees across all ages 0–17 years, and sub-populations who experienced a range of exposures, is a unique and essential tool for understanding the long-term health effects of the 9/11 attacks on exposed children and youths. The vulnerability of children makes it important to follow them to observe both short- and long-term respiratory and other effects. The fact that the event was discrete in time provides an opportunity to follow the course of healing of respiratory and psychological trauma. Ongoing follow-up of this cohort will elucidate recovery and sequelae of respiratory and toxic exposures in children.

## Figures and Tables

**Table 1:** Demographic and exposure characteristics of 3,184 children enrolled in the WTCHR, September 2003 through November 2004 [no. (%)].

Category	Resident	Nonresident student	Neither^a^	Total
Total	2,196 (69.0)	834 (26.2)	154^a^ (4.8)	3,184 (100)
Sex				
Boys	1,098 (50.0)	394 (47.2)	69 (44.8)	1,561 (49.0)
Girls	1,098 (50.0)	440 (52.8)	85 (55.2)	1,623 (51.0)
Age on 9/11 (years)				
< 5	742 (33.8)	95 (11.4)	38 (24.7)	875 (27.5)
5–11	906 (41.3)	157 (18.8)	9 (5.8)	1,072 (33.7)
12–17	548 (25.0)	582 (69.8)	107 (69.5)	1,237 (38.9)
Race/ethnicity				
White (non-Hispanic)	1,033 (47.0)	371 (44.5)	92 (59.7)	1,496 (47.0)
Hispanic or Latino	448 (20.4)	143 (17.2)	28 (18.2)	619 (19.4)
Asian	416 (18.9)	173 (20.7)	16 (10.4)	605 (19.0)
Black (non-Hispanic)	155 (7.1)	98 (11.8)	7 (4.6)	260 (8.2)
Multiple races/other	144 (6.6)	49 (5.9)	11 (7.1)	204 (6.4)
Interview type				
Proxy	2,003 (91.2)	441 (52.9)	59 (38.3)	2,503 (78.6)
Self	193 (8.8)	393 (47.1)	95 (61.7)	681 (21.4)

a This category consists of 74 youths who worked in rescue/recovery and 80 passers-by south of Chambers Street on the morning of 11 September 2001.

**Table 2:** Symptoms and conditions that began or worsened after 9/11, by age on 9/11, among 3,184^a^ children enrolled in the WTCHR, September 2003 through November 2004 [no. (%)].

	Age on 9/11 (years)			
Symptom/characteristic	0–4	5–11	12–17	Total
No.	875^a^	1,072^a^	1,237^a^	3,184^a^
Injuries on 9/11				
Eye^b^	160 (18.3)	246 (23.0)	283 (22.7)	689 (21.6)
Any other injury^c^	16 (1.8)	24 (2.2)	54 (4.4)	94 (3.0)
Symptoms that began or worsened after 9/11				
Any respiratory symptoms	441 (50.4)	574 (53.5)	665 (53.8)	1,680 (52.8)
Shortness of breath	192 (22.6)	253 (23.9)	333 (27.3)	778 (24.9)
Cough	291 (33.8)	287 (27.1)	303 (24.8)	881 (28.1)
Sinus problems	278 (32.9)	391 (37.7)	419 (34.7)	1,088 (35.2)
Throat irritation	198 (23.4)	329 (31.5)	324 (26.7)	851 (27.4)
Wheeze	246 (28.8)	273 (25.8)	278 (22.8)	797 (25.5)
Heartburn	26 (3.1)	79 (7.5)	100 (8.2)	205 (6.6)
Ever been told by a physician that you [your child] has asthma				
Before 9/11	55 (6.4)	151 (14.2)	211 (17.2)	417 (13.2)
New asthma diagnosis after 9/11	93 (10.8)	53 (5.0)	34 (2.8)	180 (5.7)
Met criteria for post-traumatic stress symptoms (“yes” to 6/8 stress questions)^d^	10 (1.2)	37 (3.7)	30 (5.3)	77 (3.2)

a Denominators are children in the age category for whom the interview provides an answer to the particular question. Some interviews are missing answers to some questions.

b Most had self-limited eye irritation from particulates in the dust cloud (five missing).

c Other injuries: sprain/strain (46), cut/laceration (48), burn (13), broken bone (3), concussion (6).

d Includes individuals < 18 years of age at interview. For individuals answering six or seven of the eight questions, we imputed answers.

**Table 3:** Prevalence of asthma before 9/11 and 2–3 years after 9/11, among 3,184 children enrolled in the WTCHR, compared with the 2003 NHIS.^a^

	WTCHR interview				NHIS 2003 survey^a^			
	Before 9/11, by age on 9/11 (*n* = 3,184)		At time of interview, by age at interview (*n* = 2,570^b^,^c^)		All children		Children from the U.S. Northeast only^d^	
Age group (years)	No.	Percent reporting asthma (95% CI)	No.	Percent reporting asthma (95% CI)	No.	Percent reporting asthma (95% CI)	Percent reporting asthma (95% CI)
0 to < 2^e^	344	2.3 (1.2–4.5)	NA^e^	NA^e^	1,434	5.0 (3.8–6.2)	5.7 (2.7–9.3)
2 to < 5^e^	522	9.0 (6.8–11.8)	399	16.0 (12.8–20.0)	2,077	9.1 (7.8–10.2)	7.0 (4.3–9.7)
5–11	1,065	14.2 (12.0–16.2)	1,143	18.7 (16.6–21.1)	4,470	14.0 (13.0–15.0)	16.8 (14.3–19.7)
12–17	1,228	17.2 (15.2–19.4)	1,005	19.0 (16.7–21.5)	4,268	14.6 (14.0–16.0)	16.6 (10.3–23.7)
Total	3,159^b^	13.2 (12.0–14.4)	2,570	18.3 (16.8–19.8)	10,692	12.4 (11.8–13.0)	14.1 (13.5–15.7)

NA, not applicable; no children were < 2 years of age at the time of WTCHR interview.

a Centers for Disease Control and Prevention (2003).

b We did not include interviews missing information on asthma diagnosis.

c We did not include 614 individuals ≥ 18 years of age at the time of the interview. NHIS children’s data are available for comparison through age 17 years.

d Reported rates of childhood asthma are higher in the Northeast than elsewhere in the United States. The NHIS Northeast group includes Maine, Vermont, New Hampshire, Massachusetts, Connecticut, Rhode Island, New York, New Jersey, and Pennsylvania.

e We divided the 0–4 age group to see the change in prevalence in the 2- to 4-year-old group.

**Table 4:** Factors associated with reporting a new diagnosis of asthma, among 3,184 children enrolled in the WTCHR, September 2003 through November 2004.

Factor	No. (%)	Percent new asthma after 9/11	Crude OR (95% CI)	Adjusted OR^a^ (95% CI)
Demographic characteristic				
Age on 9/11 (years)				
< 5	865 (27.4)	10.8	4.2 (2.8–6.3)	4.6 (3.0–7.0)
5–11	1,063 (33.7)	5.0	1.8 (1.2–2.9)	2.0 (1.3–3.1)
12–17	1,226 (38.9)	2.8	1.0	1.0
Sex				
Male	1,542 (48.9)	6.8	1.0	1.0
Female	1,612 (51.1)	4.7	0.7 (0.5–0.9)	0.7 (0.5–0.9)
Race/ethnicity				
White (non-Hispanic)	1,487 (47.2)	4.1	1.0	1.0
Hispanic	615 (19.5)	8.0	2.0 (1.4–3.0)	2.1 (1.4–3.2)
Asian	597 (18.9)	7.7	2.0 (1.3–2.9)	2.2 (1.5–3.3)
Black (non-Hispanic)	258 (8.2)	5.8	1.4 (0.8–2.6)	1.5 (0.8–2.7)
Multiple race/other/unknown	197 (6.3)	4.6	1.1 (0.5–2.3)	1.0 (0.5–2.0)
Eligibility category				
Resident only	1,100 (34.9)	6.9	1.0	NA
Student only	831 (26.4)	4.0	0.5 (0.4–0.8)	
Both	1,070 (33.9)	5.6	0.8 (0.6–1.1)	
Rescue/recovery or other	153 (4.9)	7.2	1.0 (0.5–2.0)	
Exposure				
Caught in the dust cloud on 9/11				
No	1,658 (53.6)	4.4	1.0	1.0
Yes (no eye irritation)	985 (31.6)	6.7	1.5 (1.1–2.2)	1.7 (1.2–2.4)
Yes (eye irritation)	456 (14.8)	8.6	2.0 (1.4–3.0)	2.2 (1.5–3.4)
Injured on 9/11 (excluding eye)				
No	3,061 (97.1)	5.6	1.0	NA
Yes	93 (3.0)	9.7	1.8 (0.9–3.7)	NA
Witnessed disturbing event on 9/11				
No	1,559 (49.4)	5.6	1.0	NA
Yes	1,595 (50.6)	5.8	1.0 (0.8–1.4)	NA
Symptoms of posttraumatic stress in the 4 weeks before interview (asked when < 18 years)				
No	2,322 (96.8)	6.6	1.0	
Yes	76 (3.2)	5.3	0.8 (0.3–2.2)	NA
Ever smoker at time of interview (≥12 years)				
No	1,497 (92.9)	2.9	1.0	
Yes	112 (7.1)	2.6	0.9 (0.3–3.0)	
No. of days spent south of Canal Street, 12 September through 20 December 2001				
Zero days^b^	269 (8.5)	6.7	1.0	
1–60	848 (26.6)	4.0	0.7 (0.4–1.2)	NA
61–100	803 (25.2)	7.1	1.3 (0.8–2.0)	
> 100^c^	955 (30.0)	6.1	1.1 (0.7–1.7)	
Not available	309 (9.7)	4.6		
Distance of residence from WTC site (feet; resident children, *n* = 2,196)				
< 3,000	892 (40.6)	5.0	0.7 (0.5–1.0)	
2,000–3,999	355 (16.2)	8.6	1.2 (0.8–2.0)	
4,000–5,000	279 (12.7)	6.6	0.9 (0.5–1.6)	NA
> 5,000	309 (14.1)	5.5	1.0	
Not available	361 (16.4)	7.7		
Distance of school from WTC site (feet; students on 9/11, *n* = 1,923)				
< 3,000	1,216 (63.2)	3.9	0.6 (0.3–0.9)	
2,000–3,999	113 (5.9)	1.8	0.2 (0.1–1.0)	
4,000–5,000	322 (16.7)	8.5	1.2 (0.7–2.1)	NA
> 5,000	261 (13.6)	5.1	1.0	
Not available	11 (.6)	40.0		

NA, not applicable.

a Variables considered for multiple logistic regression model: age on 9/11, sex, race/ethnicity, eligibility category, and caught in dust cloud on 9/11 with and without eye irritation.

b We categorized children as zero if they were residents who left on 9/11 and did not return, or returned after December 2001, or students who left and did not return.

c Children with >100 days are residents who did not evacuate and did not attend school outside of lower Manhattan.
